# Research on grape leaf classification based on optimized densenet201 model

**DOI:** 10.1371/journal.pone.0334877

**Published:** 2025-10-21

**Authors:** Jian Huang

**Affiliations:** Xijing University, Xi’an, China; Eskisehir Osmangazi University: Eskisehir Osmangazi Universitesi, TÜRKIYE

## Abstract

In the realm of plant classification, the classification of grape leaf varieties has long presented a complex challenge. Aiming to enhance the accuracy and generalization ability of grape leaf variety classification, this study proposes a novel approach that employs an optimized Densenet201 model for grape leaf classification. Initially, grape leaf images from five distinct varieties were meticulously collected to construct a comprehensive grape leaf dataset. To augment the diversity of the dataset, the parameters of data augmentation were delicately adjusted, with an increase in the rotation range, translation range, and so on. Subsequently, BatchNormalization and GlobalAveragePooling2D layers were incorporated to achieve feature normalization and pooling. Simultaneously, the parameters of the Dropout layer were optimized to effectively mitigate the issue of overfitting. Additionally, the number of neurons and layers in the Dense layer were varied to explore diverse network structures and pursue superior performance. Moreover, the parameters of the Adam optimizer were meticulously tuned to attain the optimal performance, and the model’s performance was further enhanced by extracting image features. The experimental results demonstrate that, in comparison with the densenet121, densenet169, resnet50, and densenet201 models, the optimized Densenet201 model showcases outstanding performance in grape leaf variety classification, remarkably improving the classification accuracy and generalization ability. This research provides a more efficient method for grape leaf variety classification.

## 1. Introduction

In the realm of plant classification, particularly when it comes to the classification of grape leaf varieties, a multitude of research efforts have been directed towards addressing this intricate issue. Traditional spectral analysis techniques encounter numerous obstacles in the classification of grape leaves. For instance, in the study of wood near – infrared spectral classification, Wan et al. (2023) [1] elucidated in their paper “BO - densenet: A bilinear one - dimensional densenet network based on multi - scale feature fusion for wood NIR classification” that traditional spectral analysis methods are marred by deficiencies in pre – processing, feature extraction, and modeling procedures. The BO – densenet model they developed achieved remarkable accuracy in wood classification, thereby highlighting the latent potential of deep learning in handling complex spectral classification tasks and offering valuable insights for grape leaf classification research.

Deep learning technology has witnessed an escalating and widespread application in the domain of plant classification and recognition. Koklu et al. have consistently demonstrated the distinctive advantages of deep – learning approaches across multiple research endeavors. In “Classification of rice varieties with deep learning methods” (2021) [2], they harnessed deep – learning algorithms to categorize rice varieties; in “The use of machine learning methods in classification of pumpkin seeds (Cucurbita pepo L.)” (2021) [3], they successfully accomplished the classification of pumpkin seeds; and in “Classification of Date Fruits into Genetic Varieties Using Image Analysis” (2021) [4], they completed the classification of date fruit genetic varieties. Additionally, Koklu et al. (2022) [5] conducted a study on grape leaf classification based on selected deep – level features in “A CNN - SVM Study based on selected deep features for grapevine leaves classification”, exploring the use of the CNN – SVM methodology and laying a solid groundwork for subsequent investigations. Simultaneously, within the field of medical image analysis, Zhou et al. (2022) [6] expounded in “Dense Convolutional Network and Its Application in Medical Image Analysis” that DenseNet and its derivatives have assumed a pivotal role. Their experiences in feature extraction and model optimization are highly pertinent and worthy of emulation in grape leaf classification research.

Research related to grapes encompasses a broad spectrum of aspects, with the study of grape leaves being no exception. Cantwell et al. (2022) [7] delved into the post – harvest biology and handling recommendations of fresh grapevine (Vitis vinifera L.) leaves in “Fresh grapevine (Vitis vinifera L.) leaves: Postharvest biology and handling recommendations”; Martín – Tornero et al. (2022) [8] employed fiber optic fluorescence data and chemometric techniques to geographically discriminate grape leaves and accurately determined the total polyphenol and chlorophyll contents at various vegetative stages in “Geographical discrimination of grapevine leaves using fibre optic fluorescence data and chemometrics. Determination of total polyphenols and chlorophylls along different vegetative stages”; Nzekoue et al. (2022) [9] meticulously analyzed the chemical characteristics of bioactive compounds and antioxidant activity of grape leaves in “Grapevine leaves (Vitis vinifera): Chemical characterization of bioactive compounds and antioxidant activity during leave development”. These studies have comprehensively unveiled the characteristics of grape leaves from diverse perspectives, furnishing crucial background information for the research on grape leaf variety classification underpinned by deep learning. Moreover, Pan et al. (2024) [10] carried out research on species identification of wild grape leaves using deep learning in “Research on species identification of wild grape leaves based on deep learning”, which not only enriches the existing body of knowledge but also provides a novel perspective and methodological reference for grape leaf classification research.

In prior research undertakings, Kaya and Saritas (2019) [11] achieved the identification and classification of vitreous durum wheat kernels by means of an artificial neural network leveraging the morphological, color, wavelet, and Gabor features of wheat kernels in “Towards a real - time sorting system: Identification of vitreous durum wheat kernels using ANN based on their morphological, colour, wavelet and gaborlet features”; Liu and Zeng (2018) [12] put forward SparseNet in “SparseNet: A Sparse DenseNet for Image Classification”, thereby introducing novel concepts for image classification; Cinar and Koklu (2022) [13] further augmented the methodological framework of plant classification through their research on rice variety identification in “Identification of Rice Varieties Using Machine Learning Algorithms”. Zhang et al. (2023) [14] constructed a lotus leaf pest and disease identification model predicated on an improved DenseNet and transfer learning in “Identification Model of Lotus Leaf Diseases and Pests Based on Improved DenseNet and Transfer Learning”. Their innovative ideas for enhancing DenseNet and its applications in image recognition can serve as invaluable technical references for the application of Densenet201 in this grape leaf variety classification research. This enables the present study to comprehensively evaluate the merits and demerits of different methods and explore a more optimized deep – learning model and approach tailored to grape leaf variety classification.

Deep learning has shown great potential in object detection and classification tasks in specific scenarios. Rahman et al. (2025) employed transfer learning techniques, using DETR and YOLOv7 models to detect coal miner images. By optimizing model parameters, they achieved a detection accuracy of 90% in complex environments, verifying the adaptability of pre-trained models in specific industrial scenarios [15]. Similarly, in the research on Hepatitis C prediction, Hossain et al. (2025) compared seven machine learning algorithms and found that the LGBM model combined with the SMOTE data augmentation technique could achieve an accuracy of 94.61%, providing a methodological reference for the processing of small-sample and imbalanced datasets [16]. In the field of multimodal signal analysis, Podder et al. (2024) converted UAV radio frequency signals into spectrograms. By optimizing the ResNet50V2 and CNN models, they improved the classification accuracy from 56.88% to 78.12% at a distance of 100 meters outdoors. Their multi-scale feature fusion strategy has inspirational significance for capturing subtle features of plant leaves [17]. For grape-related research, Subramanya et al. (2025) evaluated the performance of the EfficientNet series models in grape leaf disease classification and found that the lightweight model EfficientNetB0, with a validation accuracy of 96.73%, outperformed the more complex B5 and B7 versions, suggesting that the balance between model complexity and task adaptability is crucial [18]. Hu et al. (2025) further proposed the AF-ConvNeXt model, which integrates the Convolutional Block Attention Module (CBAM) and Feature Pyramid Network (FPN), achieving a test accuracy of 99.93% in grape leaf disease recognition, confirming the significant effect of the attention mechanism in enhancing the discriminability of key features [19].

Notwithstanding the progress made in related research, the current deep – learning investigations on grape leaf variety classification remain relatively scarce. Owing to the similar morphological characteristics of grape leaves and the profound influence of environmental factors, accurately determining grape leaf varieties poses significant challenges. This study is designed to bridge this knowledge gap. By substituting MobileNetv2 with Densenet201 in the original method and capitalizing on the latter’s architectural superiority, our objective is to enhance the accuracy of grape leaf classification. We anticipate that this research will offer robust technical support for relevant fields such as grape cultivation and agricultural production, thereby contributing to the advancement of the agricultural industry.

## 2. Research methods

### 2.1. Dataset

**Fig 1:**
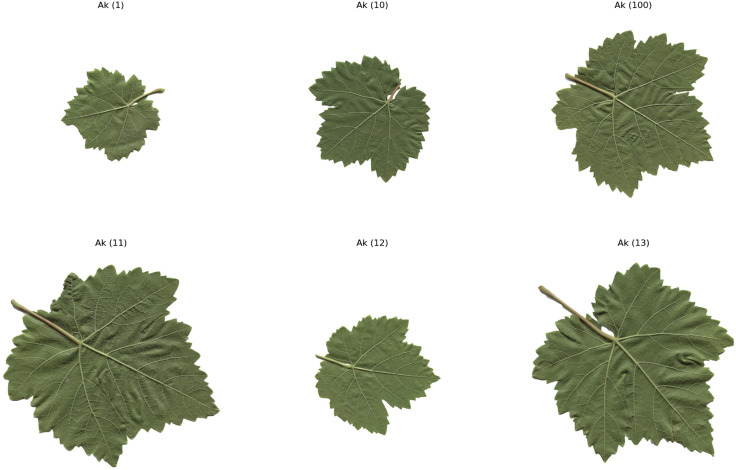
Grape leaf variety of Ak.

**Fig 2:**
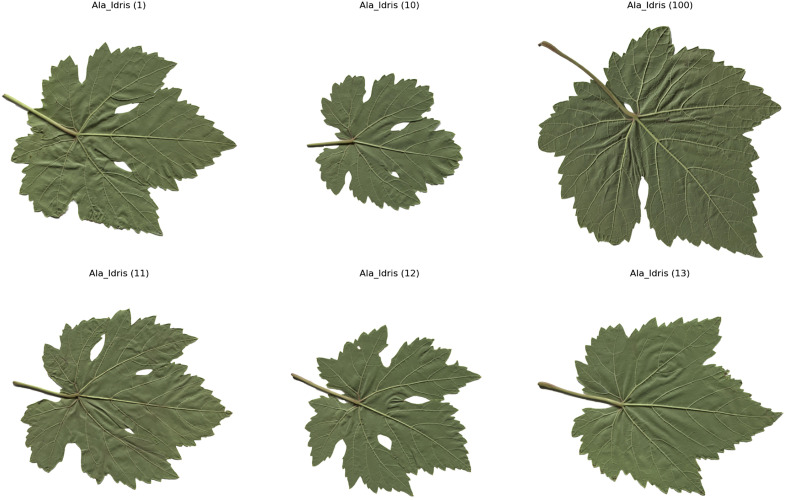
Grape leaf variety of Ala_idris.

**Fig 3:**
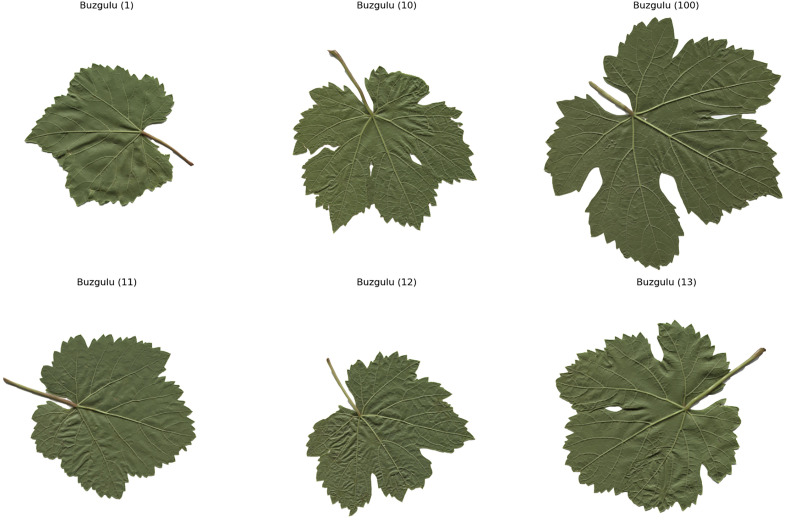
Grape leaf variety of Buzgulu.

**Fig 4:**
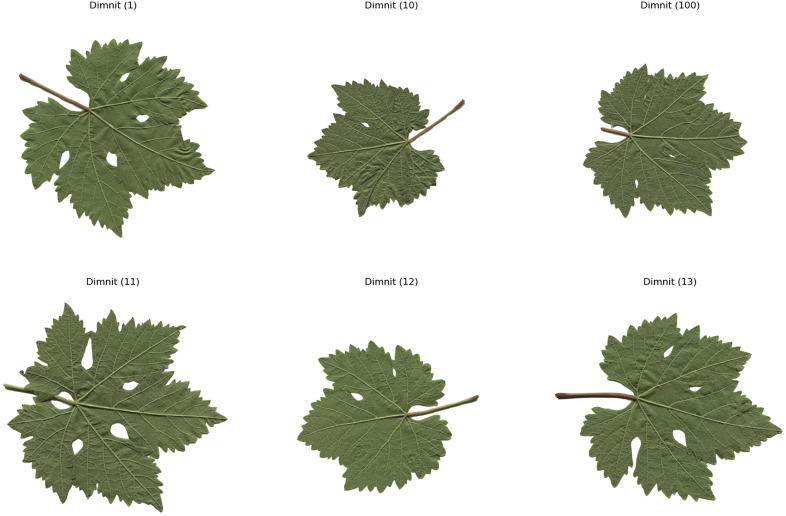
Grape leaf variety of Dimnit.

**Fig 5:**
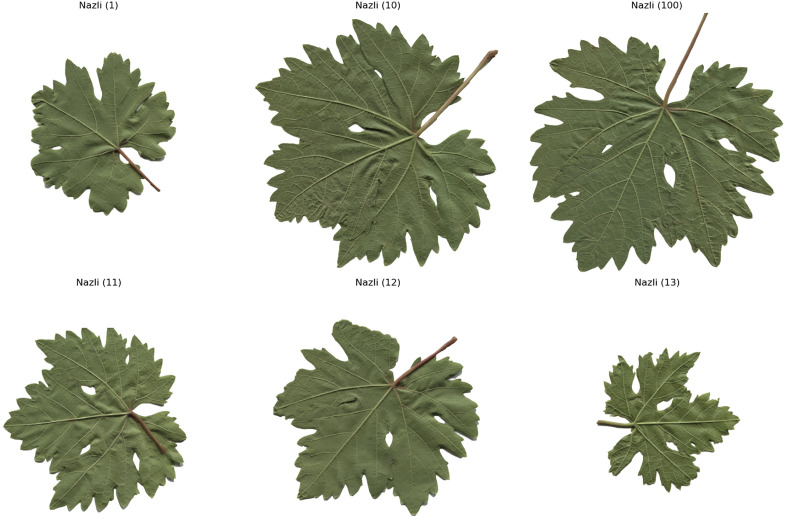
Grape leaf variety of Nazli.

The computer – vision system utilized in this research is composed of a Prosilica GT2000C camera, a camera lens mount, and a specialized illumination box. This configuration ensures uniform illumination and effectively minimizes shadows [20]. A total of 100 images for each of the five grape – leaf varieties, namely Akk, Ala Idrisi, Buzgulu, Dimrit, and Nazli, were captured prior to grape harvesting. These images were organized into a folder structure, with a total of five folders created, each dedicated to storing images of a distinct variety. To facilitate the segmentation of the leaves, the background color of all images was set to white [21]. Fig 1–5 presented hereinafter respectively exhibit the images of the five different types of grape – leaf varieties, and each image within these figures is randomly selected from the corresponding set of 100 images.

### 2.2. Data augmentation

In this study, data augmentation techniques, including rotation, scaling, and translation, were employed to expand the scale of the dataset. Each of the 100 images of each variety underwent augmentation processing, generating 400 new samples. Eventually, each variety had a total of 500 images, and the entire dataset consisted of 2,500 images. The images were resized to 224 × 224 pixels to meet the input requirements of the model. In the case of missing backgrounds, filling operations were carried out [22].

The images were normalized, with pixel values converted from 0–255–0–1. Data augmentation was further implemented, involving a rotation range of 45°, a width shift range of 0.4, a height shift range of 0.4, a shear range of 0.4, a zoom range of 0.4, and horizontal flipping. These operations were aimed at enriching the diversity of the dataset and enhancing the generalization ability of the model.

### 2.3. Optimization of Densenet201 model

Densenet201, a pre – trained convolutional neural network (CNN) model, is renowned for its dense connections between layers. This connection pattern enables efficient feature reuse and mitigates the problem of gradient vanishing. After data augmentation, improvements were made to this model. The Softmax layer of Densenet201 was adjusted to output five – class results corresponding to five grape leaf varieties. All images were resized to the input size suitable for Densenet201, typically 224 × 224 pixels. Eighty percent of the 2500 images in the dataset were used for training, and 20% were used for testing. Based on preliminary experiments, training parameters were carefully selected. The execution environment was set to GPU, the maximum number of training epochs was set to 5, the initial learning rate was 0.001, the learning rate decay factor was 0.1, the mini – batch size was 32, and the Stochastic Gradient Descent with Momentum (SGDM) optimization algorithm was adopted.

**Fig 6:**
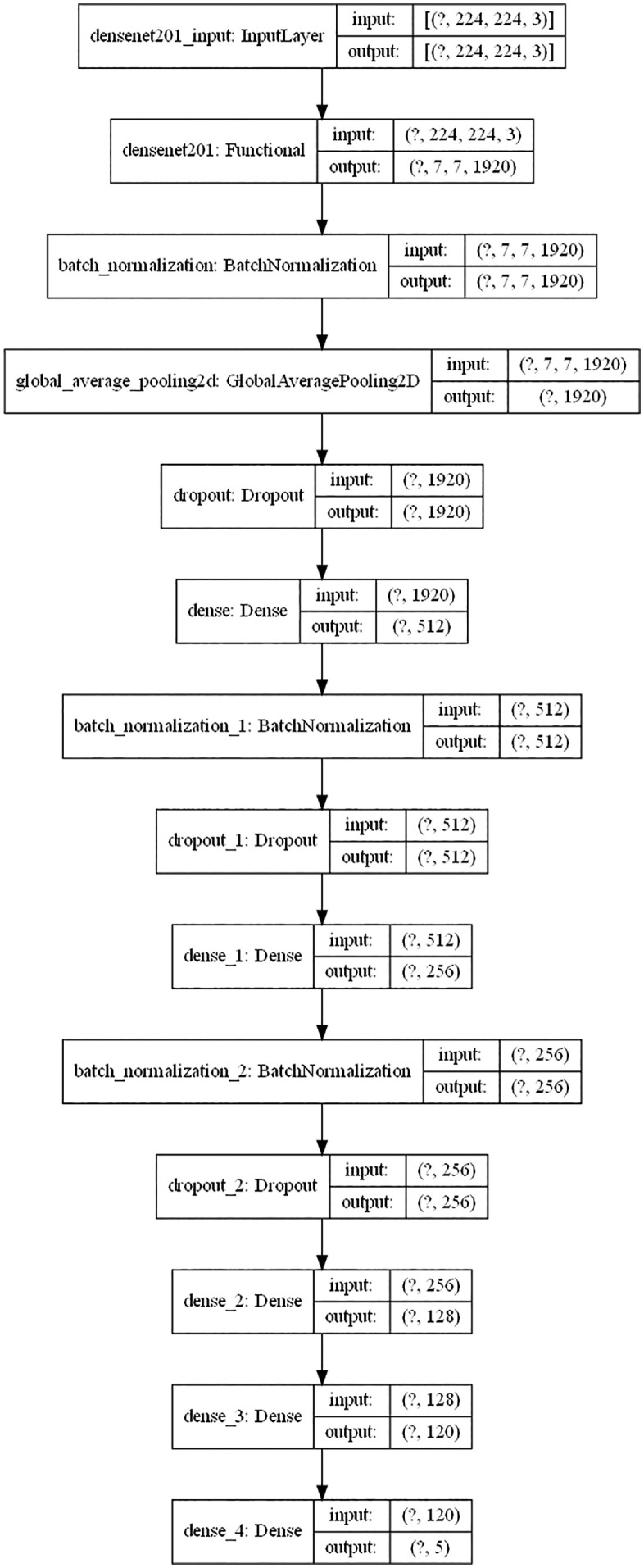
Optimized DenseNet architecture diagram.

Densenet201 has demonstrated outstanding performance in fields such as image classification. Its unique architectural design confers significant advantages in feature reuse and gradient propagation. The optimized architecture is shown in Fig 6. There are dense connections between the layers of DenseNet, meaning that each layer in the network is directly connected to all other layers. This connection method can effectively reuse features and reduce the problem of gradient vanishing. In traditional convolutional neural networks, information can usually only be transmitted between adjacent layers. However, the dense connections in DenseNet break this limitation, allowing each layer to receive the feature maps from all previous layers, greatly enhancing the efficiency of feature propagation and utilization.

In Fig 6, “?” represents the number of training epochs, and its value range is from 1 to 100. For example, “(?, 224, 224, 3)” indicates that the shape of the input data is (batch size, image height 224, image width 224, number of channels 3). Here, the batch size is undetermined, while the height, width, and number of channels of the image are fixed. A detailed description of this architecture is presented as follows:

(1) Feature extraction

Firstly, the adjusted grape leaf images with a pixel size of 224 × 224 are fed into the optimized DenseNet201 model as inputs. This model is pre – trained on the ImageNet dataset. During its construction, the top fully – connected layer is excluded (i.e.,include_top = False). DenseNet201 inherently possesses a powerful feature extraction capability, which enables it to automatically learn various features within grape leaf images. In the code, the model is loaded for feature extraction using the commandDenseNet201(weights = ‘imagenet’, include_top = False, input_shape=(224, 224, 3)).

After a series of operations such as convolution in DenseNet201, the output feature maps have a size of (7, 7, 1920). These feature maps contain rich feature information of grape leaf images, which serves as a foundation for subsequent classification tasks.

(2) Classification Process

#### 2.3.1. Batch normalization.

First, batch normalization is applied to the features extracted by DenseNet201. This operation stabilizes the feature distribution, which is conducive to the training and generalization of the model. In the code, theBatchNormalization()layer is used to implement this step. Batch normalization normalizes the input features across the mini – batch, reducing the internal covariate shift. This not only speeds up the training process but also makes the model more robust to different initializations and input distributions.

#### 2.3.2. Global average pooling.

Following the batch normalization, global average pooling is performed. This step converts the feature maps into feature vectors. By taking the average value of each feature map, the dimensionality of the features is reduced while the main feature information is retained. This helps to avoid overfitting, as it reduces the number of parameters in the model. In the code, theGlobalAveragePooling2D()function is used to achieve this operation. Global average pooling provides a simple and effective way to summarize the spatial information in the feature maps, making the model more compact and interpretable.

#### 2.3.3. Dropout.

After global average pooling, a Dropout layer is added. Neurons are randomly discarded with a certain probability (e.g., 0.4) to prevent overfitting. Dropout acts as a form of regularization by randomly “dropping out” neurons during training, forcing the model to learn more robust features. In the code,Dropout(0.4)statements are used to implement Dropout operations at different positions. This helps the model to generalize better by reducing the co – adaptation between neurons.

#### 2.3.4. Fully – connected layers.

Subsequently, a series of fully – connected (Dense) layers are used to further process and classify the features. The number of neurons in the fully – connected layers is set to 512, 256, 128, and 120 successively. The ReLU activation function is employed in these layers to introduce non – linearity into the model. Non – linear activation functions are crucial for the model to learn complex patterns and relationships in the data. For example,Dense(512, activation = ‘relu’)is used to define these fully – connected layers. Each layer transforms the input features into a new feature representation, gradually extracting more abstract and discriminative features.

#### 2.3.5. Final classification layer.

The last fully – connected layer uses the softmax activation function. This function converts the output into a probability distribution over the classes. The output dimension is set to 5, indicating the probability that a grape leaf belongs to one of the five classes. In the code,Dense(5, activation = ‘softmax’)is used to achieve the final classification output. The softmax function ensures that the output values are between 0 and 1 and sum up to 1, making it suitable for multi – class classification problems.

During the entire model training process, a custom Adam optimizer (Adam(lr = 0.0001, beta_1 = 0.9, beta_2 = 0.999)) was also employed to adjust the weights of the model. The model was trained by minimizing the categorical cross – entropy loss function (loss = ‘categorical_crossentropy’) and using accuracy (metrics = [‘accuracy’]) as the evaluation metric. Meanwhile, a learning rate scheduler (ReduceLROnPlateau) was defined. When the accuracy ceased to improve, the learning rate was decreased by a certain factor (e.g., 0.2) to facilitate better convergence of the model. Additionally, ImageDataGenerator was utilized for data augmentation to expand the dataset and further enhance the generalization ability of the model.

In this grape leaf classification task, we first performed data augmentation on the original image data to expand the diversity of the dataset and improve the generalization ability of the model. Subsequently, fine – tuning was carried out on the Densenet201 model. We adjusted the Softmax layer of Densenet201 so that it could output five types of results corresponding to the five grape leaf varieties. All images were resized to the input size suitable for Densenet201 (usually 224 × 224 pixels) to meet the input requirements of the model.

A total of 2500 images in the dataset were divided into a training set and a test set at a ratio of 80% and 20% respectively, which were used for model training and evaluation. Based on preliminary experiments, we carefully selected the training parameters to ensure the efficient and stable convergence of the model. Specifically, the execution environment was set to GPU to fully utilize its parallel computing power to accelerate the training process. The maximum number of training epochs was set to 5 to avoid overfitting. The initial learning rate was 0.001, and the learning rate decay factor was 0.1, enabling the model to converge rapidly in the early stage of training and gradually reduce the learning rate for fine – tuning in the later stage. The mini – batch size was 32. This batch training method can balance the training efficiency and the accuracy of gradient estimation to a certain extent. Moreover, we adopted the Stochastic Gradient Descent with Momentum (SGDM) optimization algorithm. The momentum term can accelerate the update of the model in the direction of the gradient, reduce oscillations, and thus find the optimal solution more quickly.

### 2.4. Model compilation

The Adam optimizer was employed with a learning rate set to 0.0001, and the values ofbeta1andbeta2were set to 0.9 and 0.999, respectively. Thecategorical_crossentropyloss function was utilized for calculating the loss in the multi – classification task. The evaluation metrics included accuracy and loss.

### 2.5. Model training

**Fig 7:**
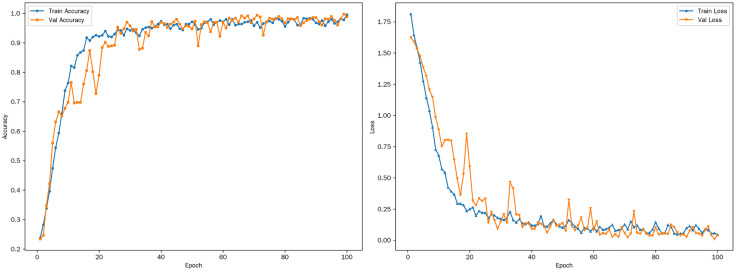
Accuracy and loss curves. **(a)** Train and Val accuracy curve **(b)** Train and Val loss curve.

The optimized model described above was employed for training, with the number of training epochs set to 100. During the training process, a custom callback function, CustomCallback, was utilized to record various metrics. CustomCallback records these metrics in a file at the end of each epoch. After all epochs are completed, it plots a curve of the metric changes. Once all training is finished, the resulting curve is as shown in Fig 7.

(1) Accuracy Curve

#### 2.5.1. Overall trend.

As can be seen from the accuracy curve in Fig 7(a), both the training accuracy and the validation accuracy show an overall upward trend as the number of training epochs progresses. This clearly indicates that the model is continuously learning and making progress, gradually being able to better capture the features and patterns in the data, thereby enhancing the accuracy of sample classification. In the initial stage of training, the accuracy rate increases relatively rapidly because the model can quickly learn the more obvious and basic features in the data. As the number of epochs increases, the rate of increase in accuracy gradually slows down, which means that the model begins to learn more complex and subtle features, and the learning difficulty increases, so the improvement amplitude becomes smaller.

#### 2.5.2. Training accuracy.

The training accuracy has been in a relatively upward state since the start of training and has maintained a relatively high level throughout the entire training process. In the initial stage of training, due to the model’s initial exposure to and learning of the training data, the accuracy rate will increase rapidly. As the training continues, the training accuracy keeps climbing and gradually approaches a relatively high value in the later stage. This indicates that the model’s fitting effect on the training data is getting better and better, and it can classify the samples in the training set more and more accurately. However, in the later stage of training, there may be some small fluctuations in the training accuracy, which may be caused by the fine – tuning of the optimization algorithm when approaching the optimal solution, or by some noise or randomness in the training data.

#### 2.5.3. Validation accuracy.

The validation accuracy also increases as the number of epochs increases. In the initial stage of training, the upward trend of the validation accuracy is similar to that of the training accuracy, but the value is lower than that of the training accuracy. This is because the validation set consists of data that the model has not seen during the training process, and its generalization ability needs some time to develop and improve. As the training progresses, the validation accuracy gradually increases, indicating that the generalization ability of the model is constantly strengthening.

#### 2.5.4. Relationship between the two.

There is a close connection between the training accuracy and the validation accuracy. Throughout the entire training process, the training accuracy is always higher than the validation accuracy, reflecting that the model’s fitting degree to the training data is better than that to the validation data. The gap between the two may be relatively large in the initial stage of training, and as the training progresses, the gap may gradually narrow, indicating that the generalization ability of the model is constantly improving.

(2) Loss Curve

#### 2.5.5. Overall trend.

In Fig 7(b), the loss value (Loss) curve clearly reveals that both the training loss (Train Loss) and the validation loss (Val Loss) display a predominantly downward trajectory. This is an auspicious indication, signifying that with the incremental increase in the number of training epochs (Epoch), the model undergoes continuous optimization, and its capacity to fit the data steadily improves.

At the onset of training, the loss value typically experiences a rapid decline. This phenomenon can be attributed to the model’s ability to swiftly discern and capture the more conspicuous features and patterns within the data during the initial learning phase. As a result, the discrepancy between the predicted outcomes and the true values is substantially reduced. However, as the training process unfolds, the rate of decrease in the loss value gradually decelerates. This is because the model transitions to learning more intricate and nuanced features, which inherently increases the learning complexity. Consequently, the marginal improvement in the loss value per training epoch becomes less pronounced.

#### 2.5.6. Training loss.

The training Loss commences at a relatively elevated level at the start of training and then precipitously drops as the number of epochs progresses. In the early training stages, the model actively learns from and adapts to the training data, rapidly identifying relatively straightforward rules that contribute to a significant reduction in prediction errors. As training advances, the training Loss continues its downward trend, gradually approaching a relatively low value.

In the latter stages of training, the training Loss may exhibit minor fluctuations. These fluctuations can be ascribed to two primary factors. First, as the optimization algorithm nears the optimal solution, it engages in fine – tuning operations in the vicinity of the local optimum. Second, the inherent noise and randomness within the training data can lead to slightly divergent learning outcomes in each iteration. Nevertheless, the overarching trend of the training Loss is one of continuous decrease, demonstrating that the model’s fitting performance on the training data is steadily enhancing.

#### 2.5.7. Validation loss.

The validation Loss also demonstrates a decreasing trend as the number of epochs increases. In the initial training phase, the validation Loss is generally higher than the training Loss. This is primarily due to the fact that the validation set comprises data that the model has not previously encountered. Consequently, the model’s generalization ability on the validation set is not yet fully developed, resulting in relatively larger prediction errors.

As the training progresses, the validation Loss gradually diminishes, which is a clear indication that the model’s generalization ability is steadily strengthening. This improvement in generalization ability reflects the model’s growing capacity to accurately predict outcomes for new, unseen data.

#### 2.5.8. Relationship between the two.

There exists an intricate and intimate connection between the training Loss and the validation Loss. Throughout the entire training process, the training Loss is generally lower than the validation Loss. This disparity reflects the model’s superior fitting performance on the training data compared to the validation data.

In the early stages of training, the gap between the two losses may be relatively substantial. However, as the training process unfolds, provided that the model possesses robust generalization capabilities, this gap will gradually narrow. A narrowing gap between the training and validation losses is an encouraging sign, suggesting that the model is effectively learning to generalize from the training data to new, unseen data.

Overall, through a comprehensive analysis of the accuracy curve and the loss curve, it becomes evident that the optimized DenseNet201 model exhibits excellent performance during both the training and validation processes. The model demonstrates robust generalization ability, enabling it to effectively accomplish the classification of grape leaves. This indicates that the optimization of the DenseNet201 model has been successful in enhancing its performance and adaptability for the specific task of grape leaf classification.

## 3. Results and discussion

### 3.1. Comparison of training and validation performance among different models

**Fig 8:**
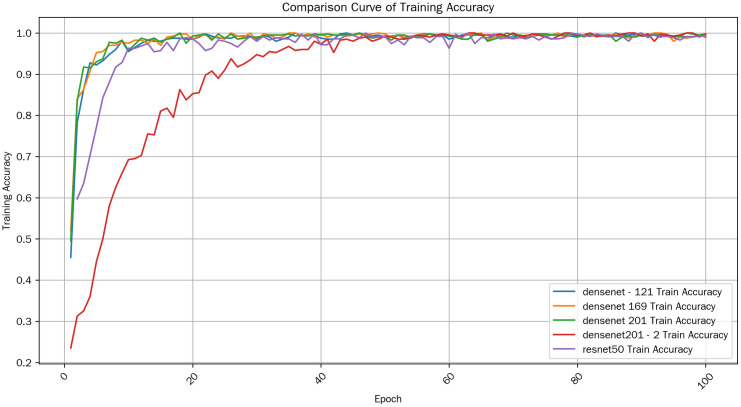
Train Accuracy Comparison.

In this study, multiple models were employed to classify the dataset. Experimental results of DenseNet121, DenseNet169, DenseNet201, the optimized model DenseNet201_2, and ResNet50 were compared. The accuracy and loss of different models on the training set and test set were analyzed and a comparative analysis was conducted. The comparison curve of Train Accuracy is shown in Fig 8.

(1) Overall Trend

From the plotted Train Accuracy curves, the training accuracies of all models generally show an upward trend with the increase of Epoch. This indicates that as the training progresses, the performance of the models on the training data becomes better and better, and they can gradually learn the patterns and regularities in the data.

(2) Comparison among Models

#### 3.1.1. Initial performance.

In the initial stage of training (when the Epoch is small), there are differences in the training accuracies of different models. For example, densenet 169 and densenet 201 have relatively high accuracies from the beginning. This may imply that their network structures are well – adapted to the data in the initial state, or their initial parameters are more conducive to quickly capturing data features. However, the initial accuracy of densenet201_2 is relatively low, perhaps because the complexity of its network structure requires more training steps to start effective learning.

#### 3.1.2. Mid – stage development.

As the Epoch advances, the growth rates of the accuracies of various models are different. Densenet 169 and densenet 201 continue to maintain a relatively stable growth and remain at a relatively high accuracy level for a certain period. Although the initial accuracy of resnet50 is not outstanding, its growth rate is considerable, and it gradually narrows the gap with the former two. This shows that resnet50 can effectively optimize itself and excavate the information in the data during the training process.

#### 3.1.3. Late – stage convergence.

In the late stage of training (when the Epoch is large), the training accuracies of some models gradually tend to be stable and approach the convergence state. For example, densenet 169 and densenet 201 have small fluctuations at a high accuracy level, indicating that the models have basically learned most of the information in the training data, and further increasing the number of training rounds has limited improvement on the accuracy. Moreover, densenet201_2 has a significant improvement in the late stage and gradually exceeds the levels of other models, indicating that it can also achieve good performance after sufficient training.

(3) Fluctuation Situation

The training accuracy curves of some models have certain fluctuations. This kind of fluctuation may be caused by random factors during the training process, such as the random gradient noise when the optimization algorithm updates the parameters, or the differences in the mini – batch sampling of the training data. For example, the curve of densenet – 121 has small – amplitude up – and – down fluctuations at some Epochs, but the overall upward trend is not affected. Generally, this kind of fluctuation is a normal phenomenon. As long as there is no significant drop in accuracy, it indicates that the model is still training stably.

**Fig 9:**
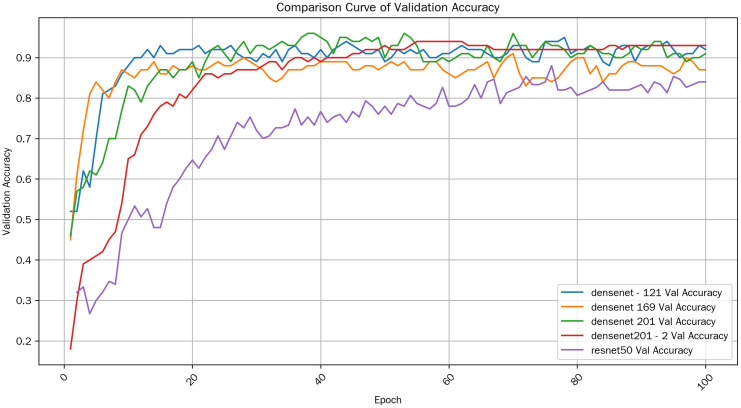
Validation accuracy comparison.

Overall, the average training accuracy of densenet201_2 in the late training stage is the highest, reaching 0.995250004529953. This indicates that on the training set, densenet 201_2 performs relatively better in the late training stage. The comparison curve of Validation Accuracy is shown in Fig 9.

(1) Overall Trend

From the perspective of the validation accuracy curves of various models, generally, as the number of training epochs increases, the validation accuracy of most models shows an upward trend. This indicates that the models are continuously learning the features and patterns in the training data and can effectively generalize this learned knowledge to the validation set.

(2) Comparison among Models
• **Initial Performance**: At the beginning of the training (low Epoch values), there are significant differences in the validation accuracy of different models. DenseNet – 121 and DenseNet 169 have relatively high validation accuracy at the initial stage. This may be attributed to the design of their network structures, which enables them to quickly capture the key features in the data. In contrast, DenseNet201_2 has a relatively low validation accuracy at the initial stage, perhaps because its network structure is more complex and requires more training steps to adjust the parameters to adapt to the data.
• **Mid – stage Development**: During the middle stage of training, the growth rates of the validation accuracy of various models vary. ResNet50 stands out in terms of its growth rate during this stage, and its validation accuracy rapidly catches up with and even surpasses that of some models with better initial performance.
• **Late – stage Convergence**: When the training progresses to the late stage (high Epoch values), the validation accuracy of each model gradually stabilizes and approaches a convergent state. However, the final convergent accuracy levels of different models are different. DenseNet201_2 achieves a relatively high validation accuracy in the late stage, indicating that it can generalize well to unseen data.

(1) Overall Trend

**Fig 10:**
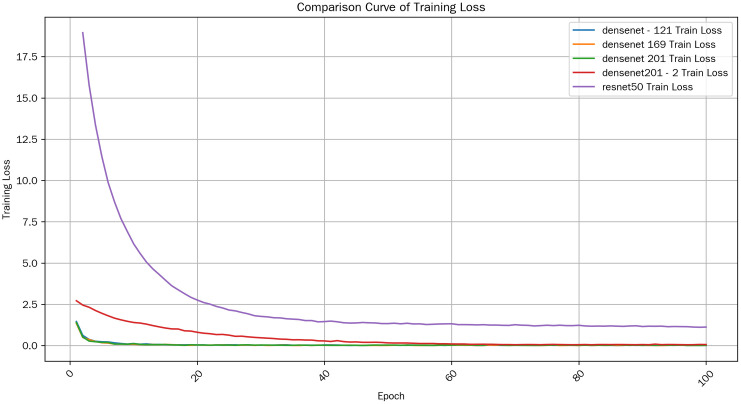
Train loss comparison.

As shown in Fig 10. From the training loss (Train Loss) curves of various models, generally, as the number of training epochs (Epoch) increases, the training loss shows a downward trend. This is in line with expectations. As the model continuously learns the features and patterns in the training data, its prediction error for the training data gradually decreases, that is, the loss value keeps decreasing. This indicates that the model is continuously optimizing its parameters to better fit the training data.

(2) Comparison among Models

#### 3.1.4. Initial performance.

At the beginning of training (low Epoch values), there are differences in the training losses of different models. The initial training losses of densenet – 121, densenet 169, and densenet 201 are relatively close, which may imply that they have similarities in model initialization or initial adaptability to the data. However, the initial loss values of densenet201_2 and resnet50 are different from the former three. The initial loss of densenet201_2 is relatively high, possibly due to its complex network structure, which makes it more difficult to adjust the parameters in the initial stage of training.

#### 3.1.5. Mid – stage development.

In the middle stage of training, the training losses of various models decrease at different rates. The training loss of resnet50 decreases at a relatively prominent rate in some stages, and it can quickly reduce the loss value. This shows that resnet50 has a strong learning ability during the training process and can rapidly capture the key features of the data and adjust the parameters. Although the decrease rates of the densenet series models are relatively stable, they are also continuously and effectively reducing the training loss.

#### 3.1.6. Late – stage convergence.

When the training progresses to the later stage (high Epoch values), the training losses of all models gradually stabilize and approach a convergent state.

**Fig 11:**
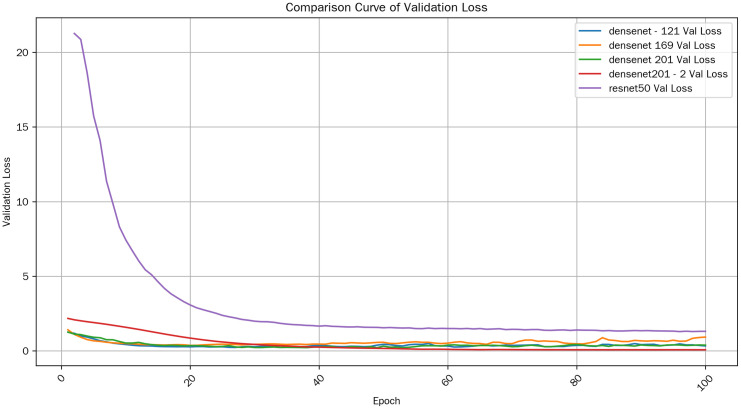
Val loss comparison.

Analyzing the validation loss curves of the models in Fig 11, we can observe that as the number of training epochs increases, the validation loss of most models generally decreases initially and then stabilizes or fluctuates within a certain range. In the early stages of training, the models have weak data – fitting capabilities. However, as they continuously learn the features and patterns in the training data, the prediction errors of the models on the validation set gradually decrease, which is manifested as a decline in the validation loss. After a certain stage of training, the performance of the models stabilizes, and the validation loss no longer shows a significant downward trend but fluctuates around a certain value.

The changing trends of the validation loss and training loss of most models are relatively reasonable, with no obvious over – fitting phenomenon. However, for the densenet201_2 model, in the early stage of training, there is a relatively large gap between the training loss and the validation loss. As the training progresses, this gap gradually narrows, indicating that its ability to control over – fitting is continuously improving, and it has a strong generalization ability.

In conclusion, compared with other models, the optimized densenet201_2 model shows an improvement in accuracy and exhibits strong generalization ability.

### 3.2. Per-class performance analysis

To delve deeper into the classification behavior of the model for varieties with similar features, this section visualizes the distribution of classification errors in different datasets (the training set, validation set, and test set) through confusion matrices. Combined with class – level precision, recall, and F1 - score, it quantitatively analyzes the causes of performance differences, with a focus on explaining the impact of feature overlap on classification results.

In the experiment, the dataset is divided into a training set (80%), a validation set (10%), and a test set (10%) at a ratio of 8:1:1. The classification model adopts an optimized DenseNet201 framework to ensure the stability and reproducibility of the results.

1) **Confusion Matrices**

**Fig 12:**
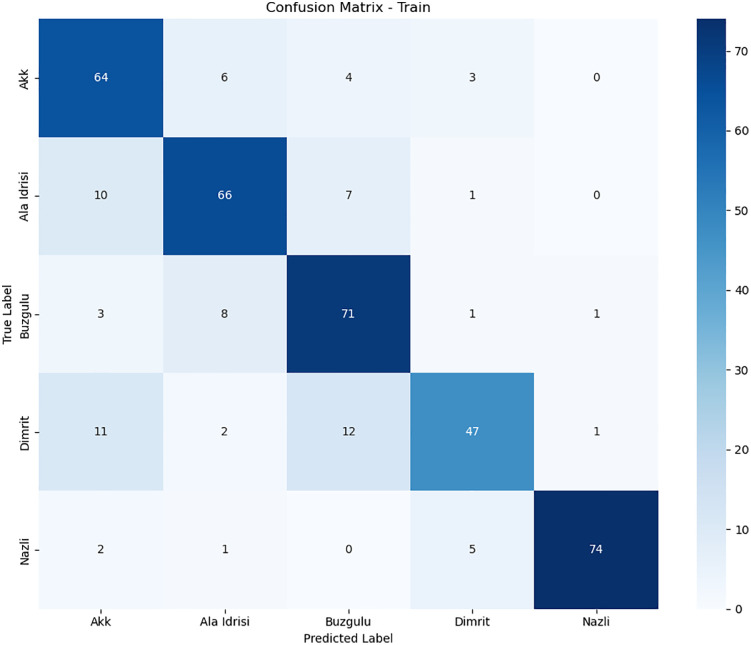
Train confusion matrix.

**Fig 13:**
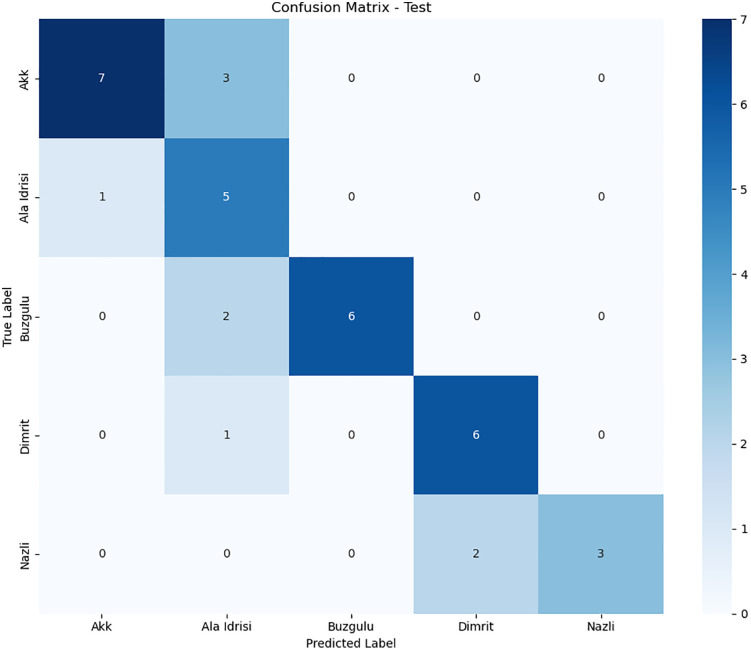
Test confusion matrix.

**Fig 14:**
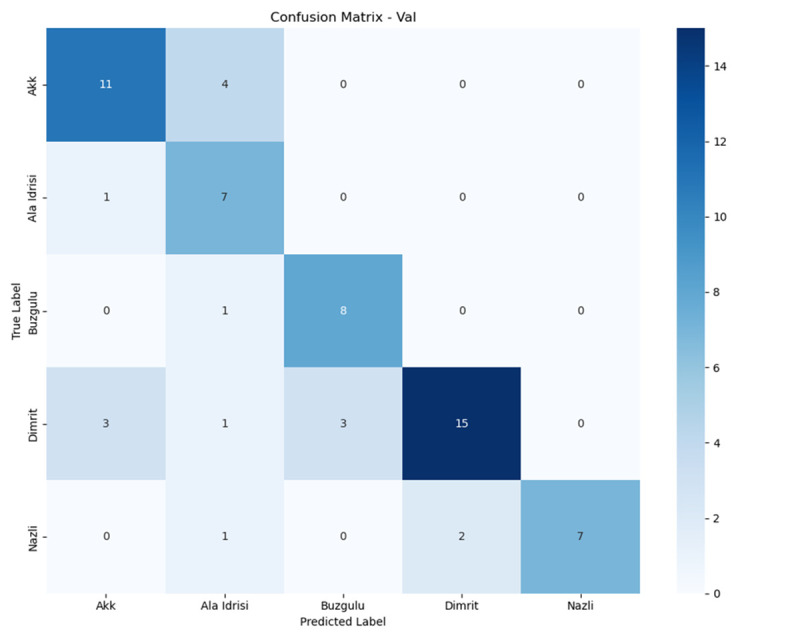
Val confusion matrix.

The confusion matrix intuitively reflects the prediction biases of the model for each category. The rows represent the true labels, and the columns represent the predicted labels. The diagonal elements are the number of correctly classified samples, and the non – diagonal elements are the number of misclassified samples. Fig 12–14 shows the heatmaps of the confusion matrices for the training set, validation set, and test set (darker colors indicate a larger number of samples).

2) **Quantitative Analysis of Class – Level Metrics**

To further quantify the performance differences among various classes, Table 1 lists the evaluation metrics of Precision, Recall, and F1 - score on the training set, test set, and validation set.

**Table 1:** Evaluation metrics of five types of grape leaves in different datasets.

Class	Precision	Recall	F1_score	source
Akk	0.88	0.7	0.78	Test Dataset
Ala Idrisi	0.45	0.83	0.59	Test Dataset
Buzgulu	1	0.75	0.86	Test Dataset
Dimrit	0.75	0.86	0.8	Test Dataset
Nazli	1	0.6	0.75	Test Dataset
Akk	0.71	0.83	0.77	Train Dataset
Ala Idrisi	0.8	0.79	0.79	Train Dataset
Buzgulu	0.76	0.85	0.8	Train Dataset
Dimrit	0.82	0.64	0.72	Train Dataset
Nazli	0.97	0.9	0.94	Train Dataset
Akk	0.73	0.73	0.73	Val Dataset
Ala Idrisi	0.5	0.88	0.64	Val Dataset
Buzgulu	0.73	0.89	0.8	Val Dataset
Dimrit	0.88	0.68	0.77	Val Dataset
Nazli	1	0.7	0.82	Val Dataset

From Table 1, we can make the following inferences:

Precision:The precision of the test set is 0.816, and that of the training set is 0.812. These two values are quite close, which means the model’s prediction accuracy for positive samples is rather similar on the test set and the training set. However, the precision of the validation set is relatively low at 0.768, indicating that its prediction accuracy is slightly worse.

Recall:The recall of the training set is the highest, reaching 0.802. The recall of the test set is 0.748, and that of the validation set is 0.776. This shows that the model can identify more positive samples on the training set, while its ability to identify positive samples on the test set is relatively weaker.

F1 - score:The F1 - score of the training set is the highest at 0.804, suggesting that the model achieves a good balance between precision and recall on the training set. The F1 - scores of the test set and the validation set are quite close, being 0.756 and 0.752 respectively, indicating that the model’s overall performance is similar on these two datasets.

In general, the model performs relatively well on the training set, while its performance on the test set and the validation set is similar but slightly inferior. This may imply that the model has a certain overfitting risk, that is, although it performs well on the training data, its generalization ability on new data (the test set and the validation set) needs to be further improved.

## 4. Discussion

Overall, the model performs well on the training set, while its performance on the test set and the validation set is slightly inferior and quite similar. This implies that the model may be at risk of overfitting, and its generalization ability needs to be enhanced. Considering that the experiment adopts an optimized DenseNet201 framework and the dataset is divided into the training set, validation set, and test set at an 8:1:1 ratio, in the future, we can consider adjusting the model structure, increasing the diversity of training data, or applying regularization methods to reduce the overfitting risk and improve the model’s generalization ability on new data.

## 5. Conclusion

This study successfully applied Densenet201 and its related methods to the grape leaf classification task. The accurate classification of grape leaves was achieved through the optimization of Densenet201. From the experimental results, in the comparison with models such as densenet121, densenet169, the optimized Densenet201 model, and resnet50, the optimized Densenet201 model demonstrated excellent performance. Meanwhile, it was also proven that this model can effectively complete the grape leaf classification task.

## Supporting information

S1 Data Test metrics. (CSV)

S2 Data Train metrics. (CSV)

S3 Data Val metrics. (CSV)
